# Does Prenatal Valproate Interact with a Genetic Reduction in the Serotonin Transporter? A Rat Study on Anxiety and Cognition

**DOI:** 10.3389/fnins.2016.00424

**Published:** 2016-09-21

**Authors:** Bart A. Ellenbroek, Caren August, Jiun Youn

**Affiliations:** School of Psychology, Victoria University of WellingtonWellington, New Zealand

**Keywords:** Autism Spectrum Disorder, latent inhibition, prepulse inhibition, novelty suppressed feeding, gene–environment interaction, animal model, SERT Knock-out

## Abstract

There is ample evidence that prenatal exposure to valproate (or valproic acid, VPA) enhances the risk of developing Autism Spectrum Disorders (ASD). In line with this, a single injection of VPA induces a multitude of ASD-like symptoms in animals, such as rats and mice. However, there is equally strong evidence that genetic factors contribute significantly to the risk of ASD and indeed, like most other psychiatric disorders, ASD is now generally thought to results from an interaction between genetic and environmental factors. Given that VPA significantly impacts on the serotonergic system, and serotonin has strong biochemical and genetic links to ASD, we aimed to investigate the interaction between genetic reduction in the serotonin transporter and prenatal valproate administration. More specifically, we exposed both wildtype (SERT^+/+^) rats and rats heterozygous for the serotonin transporter deletion (SERT^+/−^) to a single injection of 400 mg/kg VPA at gestational day (GD) 12. The offspring, in adulthood, was assessed in four different tests: Elevated Plus Maze and Novelty Suppressed Feeding as measures for anxiety and prepulse inhibition (PPI) and latent inhibition as measures for cognition and information processing. The results show that prenatal VPA significantly increased anxiety in both paradigm, reduced PPI and reduced conditioning in the latent inhibition paradigm. However, we failed to find a significant gene–environment interaction. We propose that this may be related to the timing of the VPA injection and suggest that whereas GD12 might be optimal for affecting normal rat, rats with a genetically compromised serotonergic system may be more sensitive to VPA at earlier time points during gestation. Overall our data are the first to investigate gene ^*^ environmental interactions in a genetic rat model for ASD and suggest that timing may be of crucial importance to the long-term outcome.

## Introduction

Autism spectrum disorder (ASD) is a pervasive developmental disorders that starts very early in life. In fact, most cases are diagnosed before the age of 3 and many persist into adulthood. One of the intriguing aspects of ASD is that in recent years the incidence has dramatically increased. The center for disease control and prevention in the USA found a 4-fold increase in prevalence in the period between 2000 and 2010 (http://www.cdc.gov/mmwr/pdf/ss/ss6302.pdf). Similar increases have also been reported in other countries, including Denmark (Hansen et al., 2015), Finland (Kielinen et al., 2000), the UK (Baird et al., 2006) and Australia (Williams et al., 2008). While the reasons for this increase are still hotly debated, the consequence is that the personal and economic burden of ASD is tremendous, with annual estimates as high as 175 billion US$ for the USA (Buescher et al., 2014).

Although deficits in social communication and interaction and increased repetitive and stereotyped behaviors are at the core of ASD, the majority of patients suffer from a large number of additional symptoms (Levy et al., 2009), including depression, anxiety, and cognitive deficits. While most animal modeling research has concentrated on the core symptoms, much less attention has been paid to these co-morbidities, in spite of the fact that they represent a substantial part of the clinical burden. For that reason, in this paper we aim to investigate anxiety-like symptoms and cognitive deficits in a new animal model for ASD.

Most animal models for ASD have been based on genetics (Moy et al., 2007, 2008). Indeed, a review of the SFARI (Simons Foundation Autism Research Initiative) database identified more than 70 different genetic mouse models for ASD (Banerjee-Basu and Packer, 2010). This strong focus on genetic models is not surprising given the fact that ASD was long thought to have a very high (up to 90%) heritability (Lecouteur et al., 1996). However, more recent studies have challenged this view and suggest that the heritability is closer to 50%, more similar to most other psychiatric disorders (Gauglerl et al., 2014; Sandin et al., 2014). In line with this, several (early) environmental challenges have been shown to enhance the risk of developing ASD, including prenatal stress (Kinney et al., 2008b), prenatal infections (Atladottir et al., 2010; Abdallah et al., 2012), and prenatal exposure to specific drugs, such as selective serotonin reuptake inhibitors (SSRI) (Man et al., 2015) or valproate (valproic acid, VPA) (Christensen et al., 2013). Although the risk of SSRIs inducing ASD is still controversial (Malm et al., 2016), the evidence that the mood-stabilizing and anti-epileptic drug valproate increases the risk of ASD is much stronger (Moore et al., 2000; Rasalam et al., 2005; Bromley et al., 2008; Roullet et al., 2013). For instance, in one of the most recent, large-scale, population-based studies conducted in Denmark, 4.4% of individuals prenatally exposed to VPA developed ASD, compared to 1.5% of controls not exposed to VPA - indicating a near 3-fold increased risk for ASD in those prenatally exposed to VPA (Christensen et al., 2013). In line with these findings, studies in rats and mice have shown that prenatal exposure of VPA [usually a single injection on gestational day (GD) 12] induces increases in repetitive behaviors and disruptions of social behavior (Schneider and Przewlocki, 2005; Ranger and Ellenbroek, 2015).

In contrast to the large number of animal models based on either genetic or environmental factors there is a dearth of animal models investigating an interaction between these two factors, in spite of the clinical evidence in favor of such interactions (Kim and Leventhal, 2015). In particular, several studies have investigated how the genetic risk of a variation in the serotonin transporter (SERT) is moderated by environmental factors. Serotonin (5-HT) has long been implicated in ASD, with high peripheral levels of 5-HT being one of the most replicated biochemical findings (Lam et al., 2006). In addition, reduced levels of SERT and 5-HT2A receptors have repeatedly been found in drug naïve patients (Zurcher et al., 2015). Genetic studies investigating the serotonergic system and especially the SERT have been less consistent, although several studies have suggested that genetic alterations, such as the 9/10 repeat of the STin2 and the s-allele of the 5-HTTLPR (5-Hydroxytryptamine transporter linked polymorphic region) may be linked to ASD (Nyffeler et al., 2014; Warrier et al., 2015). Several reasons for this inconsistency have been proposed including genetic heterogeneity of ASD and the possibility that these genetic variants are only related to specific (subsets of) symptoms. In addition, most genetic linkage studies have ignored the potential interaction with environmental factors. Indeed two recent studies showed that while the s-allele of the 5-HTTLPR by itself did not constitute a significant risk factor for ASD or ASD-like symptoms, the risk was significantly enhanced in combination with prenatal smoking or stress (Nijmeijer et al., 2010; Hecht et al., 2016).

Interestingly, the s-allele of the 5-HTTLPR has also been associated with anxiety (Kenna et al., 2012) and cognitive deficits (Weiss et al., 2014), which are also prominent in patients with ASD, as mentioned above. Since prenatal valproate significantly affects serotonergic neurotransmission (Miyazaki et al., 2005; Oyabu et al., 2013), the present study investigates the potential interaction between valproate and a genetic reduction of the SERT. More specifically, we injected heterozygous SERT knock-out and wildtype rats prenatally with a moderate dose (400 mg/kg s.c.) of valproate and investigated the behavior of the offspring in two models for anxiety (elevated plus maze and novelty suppressed feeding) and two models assessing cognition and information processing (latent inhibition and prepulse inhibition). To our knowledge, this study is the first which combined two well known risk factors in a rat model of autism. Given the high prevalence of the risk SERT polymorphism in the general population and the wide spread use of valproate for various conditions in pregnant mothers (Christensen et al., 2013), the relevance for our study stands to be considerable.

## Materials and methods

### Animals

The SERT knock-out (Slc6a4^1Hubr^ SERT^−/−^) rats were originally developed by us using ENU mutagenesis in Wistar rats (Smits et al., 2006; Homberg et al., 2007a). In the present study male and female SERT^+/−^ rats were mated overnight and the presence of a vaginal plug the next morning was taken as a sign of successful conception and counted as Gestational Day 1 (GD1). On GD12.5, half of the pregnant females were subcutaneously injected with 400 mg/kg of VPA (valproic acid, Sigma-Aldrich, Auckland, NZ) with the remaining half injected with saline 1 ml/kg. The dose of VPA was based on pilot studies in our own laboratory showing that this dose produced moderate effects on anxiety in normal wild-type animals. Moreover, the dose is in line with other previously published reports (Kim et al., 2011), although higher doses (up to 800 mg/kg) have also been used (Schneider et al., 2008; Dufour-Rainfray et al., 2010; Bambini-Junior et al., 2011; Oyabu et al., 2013; Raza et al., 2015). However, it is important to realize that we aimed to induce only moderate effects in wildtype animals so as to be able to observe a gene ^*^ environment interaction without the risk of inducing a ceiling effect. The treatment led to six different groups of offspring, of which only four were used: VPA/SERT^+/−^, VPA/SERT^+/+^, SAL/SERT^+/−^, and SAL/SERT^+/+^. Given that ASD is much more common in males than in females, only male offspring were used for this study. The rats were weaned at 21 days of age when a small ear punch (2 mm diameter) was taken for genotyping (genotyping was performed by Transnetyx, Cordova, USA). Males were housed in groups of 2–4 in standard housing cages in the animal facility of the School of Psychology, Victoria University of Wellington facilities. Rooms were humidity (77%) and temperature (21°C) controlled and kept on 12 h light/dark cycles (lights on 07:00 a.m.). The animals had free access to food and water except during the novelty suppressed feeding (in preparation for which they were deprived of food for 24 h prior to the experiment, see below) and the latent inhibition experiment (during which water intake was restricted, see below). All experiments were performed in adulthood (>postnatal day 60) with experimentally naïve animals. Animals from at least 4 different litters were used for every experiment to prevent any possible litter effect. All experiments were in accordance with institutional and national guidelines and were approved by the animal ethics committee of Victoria University Wellington.

### Elevated plus maze

The elevated plus maze (EPM) consisted of four arms of equal length (50 cm) and width (10 cm), with two opposing arms enclosed by opaque walls (height 20 cm, closed arms), and the two remaining arms only having a shallow raised transparent (1 cm) lip (open arms). Rats were briefly handled for 2 consecutive days prior to the start of the experiment to habituate them to pre-test stressors. Immediately before the EPM, rats were placed in an open field for 5 min to encourage exploratory behavior (Pellow et al., 1985). Immediately after the open field test, the rats were placed in one of the closed arms and the behavior was recorded for 5 min using Ethovision XT9 (Noldus, Wageningen, the Netherlands). Offline analysis was performed to assess latency to first emerge from the entry arm, frequency and duration in each arm as well as overall percentage of time spent in the open and closed arms. Arm entry was defined as the tail base point entering the arm. Total distance traveled and velocity was also recorded, along with assessment of the frequency and duration of the stretched attend posture, defined as a body posture state which is over 75% of elongation measure. Elongation is expressed as a percentage and ranges from 0 (when the subject's shape is perfectly circular) to 100% (when the subject's shape is a line). In total 67 rats were used (see Table 1).

**Table 1:** **Total number of animals used in the different experiments**.

**Group**	**EPM**	**NSF**	**LI-PE**	**LI-NPE**	**PPI**
SAL/SERT^+/+^	14	9	7	6	15
SAL/SERT^+/−^	22	11	9	9	13
VPA/SERT^+/+^	12	10	6	6	12
VPA/SERT^+/−^	19	12	9	9	12
Total	67	42	31	30	52

### Novelty suppressed feeding

The novelty suppressed feeding (NSF) test is based on the conflict between exposure to an open area and the drive to eat (Olivier et al., 2008). All animals were again handled briefly for 2 consecutive days prior to the experiment. Rats were food deprived 24 h after which they were placed next to the wall in a circular open field (80 cm diameter, with 30 cm high walls), facing the wall. A food pellet was placed in the center of the arena on a small (6 cm diameter) circular piece of filter paper. The latency to start eating was taken as the dependent variable. When animals failed to start eating within 10 min, they were removed from the open field and the analysis. In total 42 rats were included in the analysis (see Table 1).

### Latent inhibition

Latent inhibition (LI) is often used to assess selective attention (Lubow, 1989) and is based on the phenomenon that conditioning of a stimulus is retarded when that stimulus has previously been presented repeatedly without any direct consequences. In the present experiment we used the conditioned taste aversion protocol to assess LI (Ellenbroek et al., 1997). For this experiment, rats were singly housed for 24 h before the start of the experiment with water bottles removed. For 3 consecutive days rats were given a single bottle with either tap water (non-pre-exposed) or a 5% sucrose solution (pre-exposed) for 30 min and the total volume consumed was recorded. On the fourth day all rats were given 30 min access to a 5% sucrose solution after which they were intraperitoneally injected with 75 mg/kg lithium chloride (10 mg/ml, Sigma-Aldrich, Auckland, NZ). On the fifth and final day all animals were given a 30 min free access to one bottle of tap water and one bottle of 5% sucrose. The dependent variable was the percentage of sucrose over tap water consumed on the final day. In total 61 rats were used (see Table 1).

### Prepulse inhibition

Prepulse inhibition (PPI) is used to assess information processing, in particular sensorimotor gating (Braff and Geyer, 1990). PPI was assessed using San Diego Instrument startle equipment as previously described (Ellenbroek et al., 1995, 1996). The startle chamber consisted of a Plexiglas tube (diameter 8.2 cm, length 25 cm), placed in a sound-attenuated chamber, in which the rats were individually placed. The tube was mounted on a plastic frame, under which a piezoelectric accelerometer was mounted, which recorded and transduced the motion of the tube. After the rats were placed into the chamber, they were allowed to habituate for a period of 5 min during which a 70 dB[A] background noise was present. After this period the rats were exposed to five startle stimuli (120 dB[A], 20 ms in duration), followed by a series of startle and prepulse inhibition trials. Prepulse inhibition trials consisted of a prepulse (72, 74, 78 or 86 dB[A] intensity, 20 ms duration) followed 100 ms later by the same 120 dB[A] startle stimulus. The session ended with another block of five 120 dB[A] startle trials. The inter-trial interval was pseudorandom between 10 and 20 s, and the entire session lasted for about 17 min. The resulting movement of the rat in the startle chamber was measured during 100 ms after startle stimulus onset (sampling frequency 1 kHz), rectified, amplified and fed into a computer, which calculated the maximal response over the 100-ms period. Basal startle amplitude was determined as the mean amplitude of the 10 startle trials that were interspersed with the first prepulse inhibition trials. Prepulse inhibition was calculated according to the formula 100 × [1 – (PPx/P120)], in which PPx is the mean of the 10 prepulse inhibition trials (PP72, PP74, PP78 or PP86), and P120 is the basal startle amplitude. Percentage startle habituation was calculated as 100^*^P120l/P120f, with P120f, and P120l being the average of the first and the last five startle responses, respectively.

### Statistical analysis

Data were statistically evaluated using SPSS Statistics v20 using two or three way factorial Analysis of Variance (ANOVA) with *p* < 0.05 considered statistically significant. More details can be found in the results section of each experiment.

## Results

### EPM

**Figure 1:**
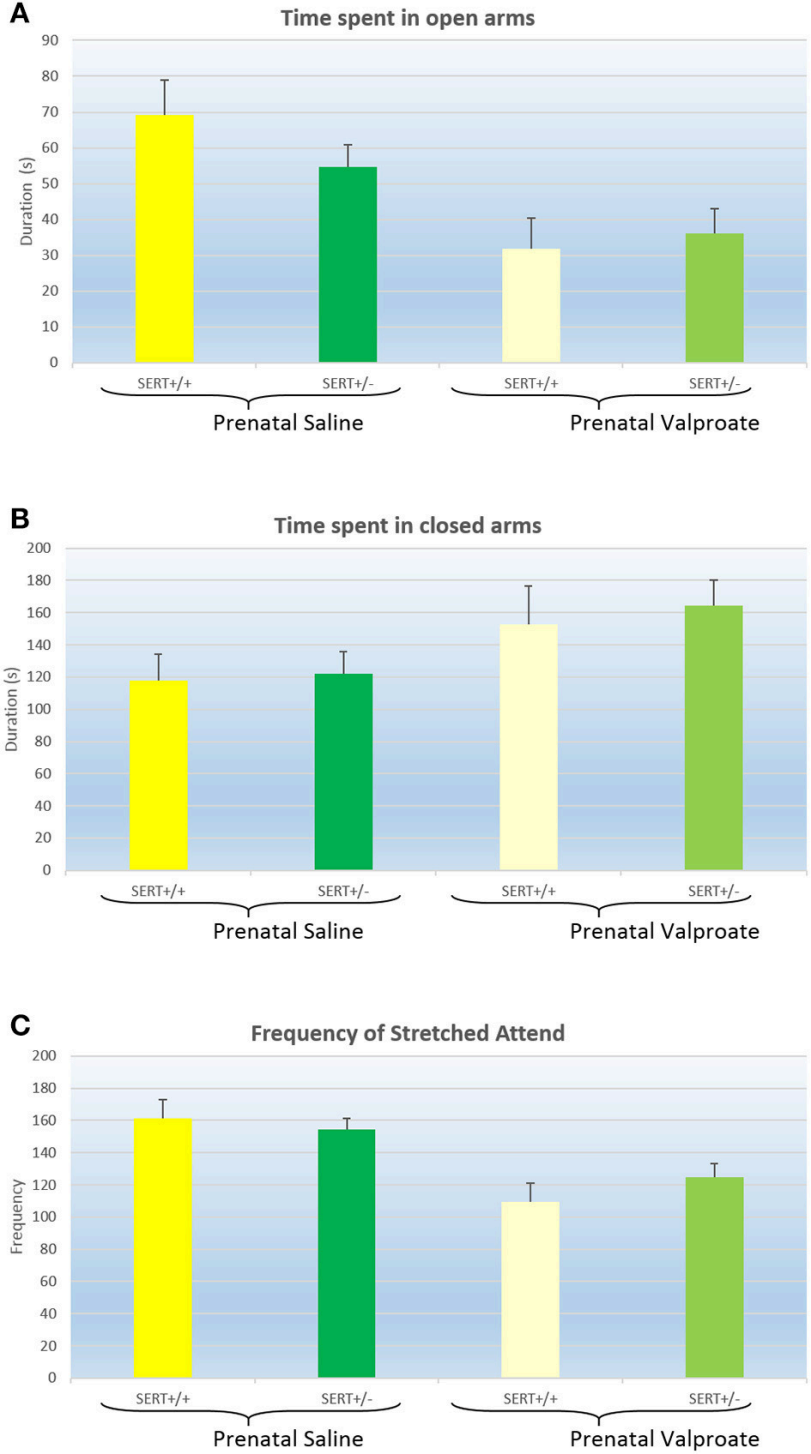
**The effects of prenatal saline or valproate exposure in wildtype (SERT^+/+^) and heterozygous SERT knockout (SERT^+/−^) rats on the elevated plus maze. (A)** Time (s) spent in the open arms; **(B)** Time (s) spent in the closed arms; **(C)** Frequency of stretched attend posture. Represented are the mean values plus Standard Error of the Mean. The number of animals in each group is shown in Table 1. Data were analyzed with a two way ANOVA with prenatal treatment and genotype as between subject factors.

The results of the EPM are displayed in Figure 1 and were analyzed with a two way ANOVA with prenatal treatment and genotype as between subject factors. Both the time spent in the open arm [Figure 1A, *F*_(1, 61)_ = 12.8, *p* < 0.001] and in the closed arm [Figure 1B, *F*_(1, 61)_ = 5.1, *p* < 0.05] showed a significant prenatal treatment effect, with VPA decreasing the time spent in the open arms (and subsequently increasing the time spent in the closed arms). However, there was no significant genotype effect, nor any interactions between genotype and prenatal treatment. The total distance moved and the average speed did not differ between the groups (see Table 2). The frequency of stretched attend posture is displayed in Figure 1C. A two way ANOVA identified a significant treatment effects [*F*_(1, 61)_ = 18.9, *p* < 0.001], while the genotype [*F*_(1, 61)_ = 0.21, ns]and interaction [*F*_(1, 61)_ = 1.43, ns]were not significant.

**Table 2:** **Total distance traveled and speed in the EPM**.

**Group**	**Distance traveled (cm)**		**Speed**	
	**Mean**	**S.E.M**.	**Mean**	**S.E.M**.
SAL/SERT^+/+^	1472.7	83.0	5.00	0.27
SAL/SERT^+/−^	1414.2	57.6	4.75	0.19
VPA/SERT^+/+^	1322.0	144.1	4.70	0.55
VPA/SERT^+/−^	1597.2	115.7	5.42	0.41

Represented are averages plus S.E.M. Data were analyzed with a two way ANOVA with prenatal treatment and genotype as between subject factors.

### NSF

**Figure 2:**
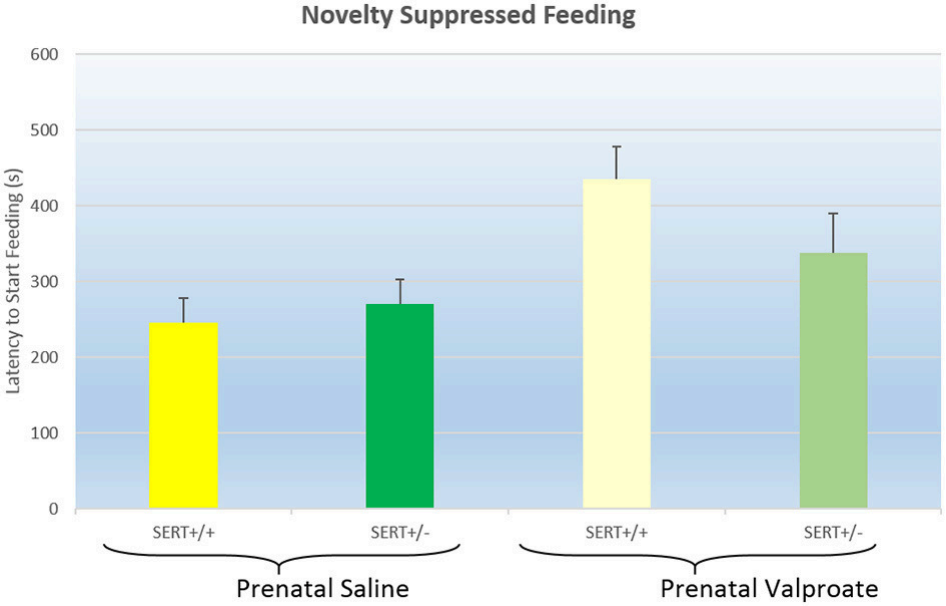
**The effects of prenatal saline or valproate exposure in wildtype (SERT^+/+^) and heterozygous SERT knockout (SERT^+/−^) rats on the novelty suppressed feeding task**. Represented are the mean latency to start eating plus Standard Error of the Mean. The number of animals in each group is shown in Table 1. Data were analyzed with a two way ANOVA with prenatal treatment and genotype as between subject factors.

The latency to start feeding in the NSF test are displayed in Figure 2. A two way ANOVA found a significant effect of prenatal treatment [*F*_(1, 38)_ = 5.2, *p* < 0.05], but not a genotype effect [*F*_(1, 38)_ = 0.2, ns] nor a significant interaction [*F*_(1, 38)_ = 2.3, *p* = 0.12]. Inspection of the figure shows that prenatal VPA exposure significantly increased the latency to eat.

### LI

**Figure 3:**
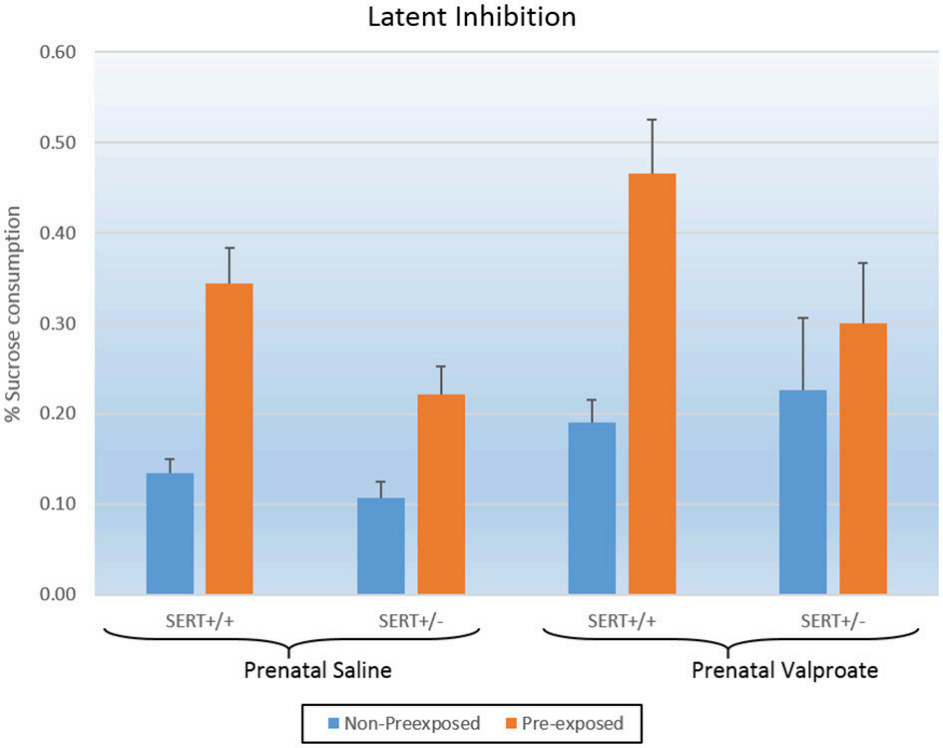
**The effects of prenatal saline or valproate exposure in wildtype (SERT^+/+^) and heterozygous SERT knockout (SERT^+/−^) rats on the latent inhibition**. Represented is the % sucrose consumption on the final day for both the animals pre-exposed and non-preexposed to sucrose for 3 days prior to conditioning. Represented are the mean values plus Standard Error of the Mean. The number of animals in each group is shown in Table 1. Data were analyzed with a three way ANOVA with prenatal treatment, genotype and pre-exposure as between subject factors.

Figure 3 shows the results of the LI experiments, which were statistically analyzed using a three way ANOVA, with prenatal treatment, genotype and exposure to sucrose as between subject factors. The analysis showed significant effects of exposure [*F*_(1, 52)_ = 20.2, *p* < 0.001] and of prenatal treatment [*F*_(1, 52)_ = 6.3, *p* < 0.02]. In addition, there was a tendency for a genotype effect [*F*_(1, 52)_ = 3.5, *p* = 0.06] as well as a significant genotype ^*^ exposure interaction [*F*_(1, 52)_ = 3.9, *p* = 0.05]. As indicated in figure 3, valproate treatment significantly increased sucrose consumption. We subsequently split the data according to genotype and found that while VPA increased sucrose consumption in SERT^+/+^ rats [*F*_(1, 20)_ = 5.3, *p* < 0.05], this was not seen in SERT^+/−^ rats [*F*_(1, 32)_ = 3.2, *p* = 0.08]. Interestingly, a close look at figure 3 shows that, in contrast to all others groups, the VPA/SERT^+/−^ group did not show significant LI effect (i.e., no statistically significant differences between pre- and non- pre-exposed animals), although the overall interaction failed to reach significance.

### PPI

**Figure 4:**
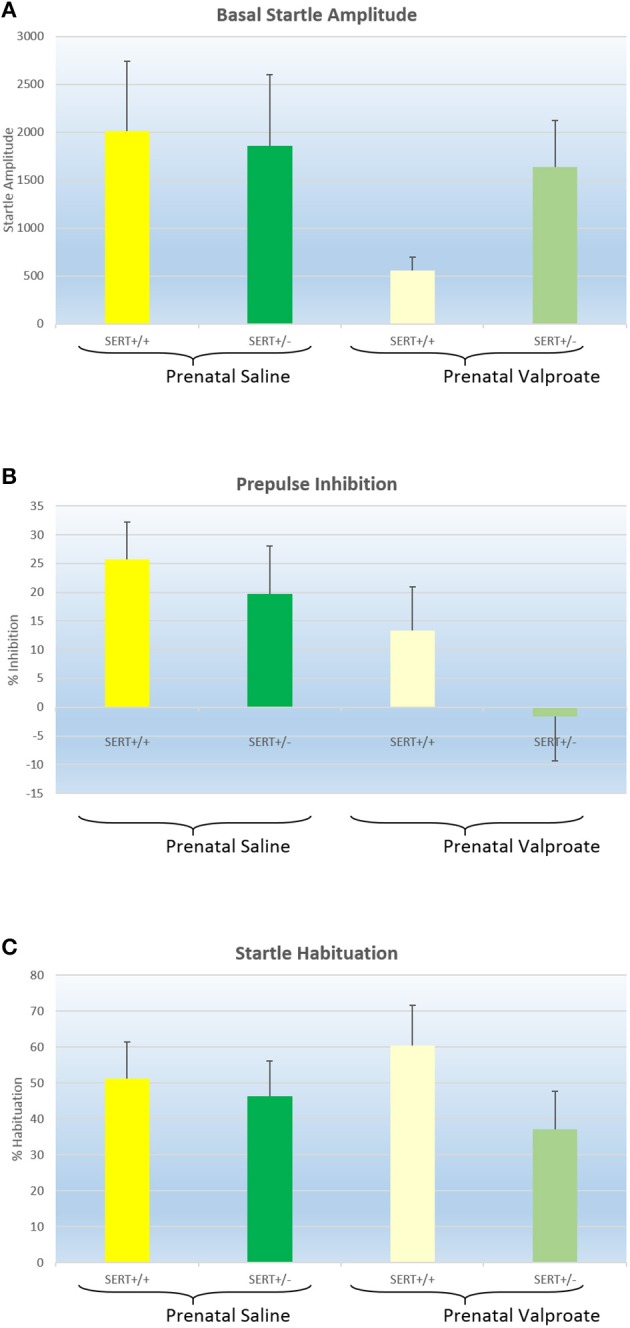
**The effects of prenatal saline or valproate exposure in wildtype (SERT^+/+^) and heterozygous SERT knockout (SERT^+/−^) rats on acoustic startle responding. (A)** Basal startle amplitude; **(B)** Percentage prepulse inhibition; **(C)** Percentage habituation. Represented are the mean values plus Standard Error of the Mean. The number of animals in each group is shown in Table 1. Data were analyzed with a two way ANOVA with prenatal treatment and genotype as between subject factors.

Figure 4A shows the basal startle amplitude of rats prenatally treated with either saline or VPA. Neither the genotype [*F*_(1, 51)_ = 0.6, *p* > 0.4] nor the prenatal treatment effects was significant [*F*_(1, 51)_ = 1.9, *p* = 0.17]. While there was a significant within-subject effect of prepulse intensity, there were no 2 or 3 way interactions between prepulse intensity, genotype and/or prenatal treatment (data not shown). Therefore, we collapsed all prepulse intensities into a single prepulse inhibition value (Figure 4B). Two way ANOVA revealed a significant effect of prenatal treatment [*F*_(1, 51)_ = 5.1, *p* < 0.05] with reduced PPI in the valporate group, but there was no effect of genotype nor an interaction. Startle habituation, on the other hand (see Figure 4C), was not significantly affected by either genotype or prenatal treatment.

## Discussion

Valproic acid (VPA) is a well-established risk factor for the development of ASD in humans (Christensen et al., 2013), and subsequently, multiple studies in mice and rats have shown that a single injection of VPA to pregnant dams can induce ASD-like symptoms. These include deficits in the core aspects of ASD: social behavior, communication and stereotypy (Schneider et al., 2008; Roullet et al., 2013; Ranger and Ellenbroek, 2015), although differences exist between individual studies in relation to dose, route and timing of administration as well as species. In line with our current results, valproate has also been found to increase anxiety (Schneider et al., 2008; Mehta et al., 2011), and decrease PPI (Schneider and Przewlocki, 2005), although the latter is not uniformly found (Dendrinos et al., 2011). To the best of our knowledge, this paper is the first to evaluate the effect of VPA in a model of LI. Deficits in cognition have also been previously reported in rats prenatally exposed to VPA, including reduced alterations in the Y-maze (Markram et al., 2008), and alterations in the Morris water maze (Frisch et al., 2009). Given that there was no significant interaction between prenatal treatment and sucrose pre-exposure, the significant effect of VPA needs to be interpreted as an overall impairment in conditioning as seen in the global increase in sucrose consumption (see Figure 3), rather than a specific decrease in LI.

Both the EPM and the NSF showed that prenatal VPA increased anxiety-like behavior, as seen by a decrease in time spent in the open arms in the EPM and an increase in the latency to start feeding in the NSF. Importantly, in the EPM there was no influence of VPA treatment on total distance traveled or average speed, suggesting that the observed differences in time spent are not secondary to differences in exploratory behavior. Intriguingly, we found that VPA significantly reduced the frequency of stretched attend posture (as well as the total duration, data not shown). This was unexpected as stretched attend posture is usually considered a sign of anxiety. Thus, in most studies a decrease in time spent in the open arms is accompanied by an increase in stretched attend postures (Cole and Rodgers, 1994; De Almeida et al., 1998; Fodor et al., 2016). However, a more detailed analysis suggests that stretched attention and time spent on the open arm are not necessarily strongly related to each other. For instance, Semaphorin 5a mutant mice (proposed as another animal model for ASD) show no difference in time spent on the open arms but a significantly increased frequency of stretched attend posture (Gunn et al., 2011). Perhaps more importantly, in a detailed analysis of anxiety like behavior in mice strains, factor analysis showed that stretched attend postures and time spent on the open arms loaded on independent factors (O'leary et al., 2013). In a similar vein, an analysis of the Roman high and low avoidance strains found that while differences in stretched attend postures were predictive of performance in an active avoidance paradigm, frequency of open arm entry was predictive of fear potentiated startle. Together these data suggest that the relationship between these two variables is far from clear and it has indeed been suggested that a stretched attend posture is more related to risk-assessment than to anxiety *per se*. Analysing risks in a novel environment is a crucial part in survival in both animals and humans with a complicated interaction between emotion (anxiogenic environment) and cognition (risk assessment). Hence, it might be speculated that decreased stretch posture might indicate an impairment in attention and active exploration required in successful adaptation to a novel surroundings. This is reminiscent of the less active engagement with stimuli and insistence on sameness in a novel environment in autistic children (Eisenberg et al., 2015).

The current study found virtually no genotype effects, except for a tendency in the LI experiment (*p* = 0.06). Although this might be somewhat surprising, given the role 5-HT and the SERT play in, especially, anxiety, it is important to keep in mind that we used rats heterozygous for the SERT ablation. Previous experiments from our lab found that homozygous SERT knock out rats show a significant decrease in time spent on the open arms of the elevated plus maze as well as an increased latency to start feeding in the novelty suppressed feeding task (Olivier et al., 2008), consistent with an anxious phenotype. A reduction in latent inhibition has also previously been reported for homozygous SERT knock out rats (Nonkes et al., 2012), which was thought to be, at least in part, responsible for the increased performance in a set-shifting task. Previous research from our group has implicated the basal ganglia in latent inhibition as measured using the conditioned taste aversion paradigm (Ellenbroek et al., 1997), although other structures, including part of the prefrontal cortex are also likely involved (Nonkes et al., 2012). Pharmacologically, most studies on latent inhibition have focussed on dopamine (Moser et al., 2000). There is also evidence that 5-HT is involved, although the exact mechanism is unclear (Moser et al., 2000). Thus, while both 5-HT1A and 5-HT3 receptor antagonists facilitate LI (Warburton et al., 1994; Killcross et al., 1997), selective serotonin reuptake inhibitors induce the same effect (Moser et al., 2000), thus suggesting a complicated relationship between serotonin transmission and LI.

Interestingly, none of the experiments of the current series found a significant gene–environment interaction. Inspection of Figure 3 suggests that in contrast to all groups, the SERT^+/−^ rats prenatally treated with valproate did not show a significant LI (indeed this group was the only one that did not show a significant difference between the pre-exposed and non-pre-exposed). However, as the prenatally saline treated SERT^+/−^ group also had a smaller LI, compared to SERT^+/+^ group, the interaction did not reach significance.

The lack of any significant gene–environment interaction is surprising, given the prominent role 5-HT plays in ASD as well as the studies in rats and mice that have shown that prenatal valproate affects the serotonergic system. Previous studies have shown that prenatal valproate increases 5-HT levels in the frontal cortex (Narita et al., 2002; Tsujino et al., 2007) and decreases these levels in the hippocampus (Dufour-Rainfray et al., 2010), while not altering SERT levels within the brain (Dufour-Rainfray et al., 2010). Intriguingly, studies have also found a significant effect of VPA on 5-HT development, with prenatal VPA treatment leading to a caudal shift in 5-HT cell bodies within the brain (Miyazaki et al., 2005). *In vitro* research suggests that this may be due to a retarded neuronal maturation, possibly mediated via an effect on Sonic Hedgehog (a protein involved in serotonergic development). Subsequent studies from the same group confirmed a reduction in mRNA for Sonic Hedgehog at GD11.5, associated with an abnormal distribution of 5-HT neurons in the caudal rostral raphe nucleus (Oyabu et al., 2013).

These convincing biochemical and morphological data beg the question why we failed to find (strong) evidence for a gene–environment interaction. Several different possible explanations can be put forward. The most parsimonious explanation is that these biochemical findings simply do not translate into ASD-like behavioral symptoms. However, this seems unlikely given the strong association between 5-HT alterations and ASD symptoms in humans (Lam et al., 2006). Moreover, rats and mice with a complete lack of the SERT show ASD-like features (Homberg et al., 2007b; Moy et al., 2009). Likewise, transgenic mice with another point mutation in the SERT (Ala56, also leading to hyperserotonemia), also show deficits in the core symptoms of ASD (Veenstra-Vanderweele et al., 2012). Finally, as mentioned in the introduction, there is increasing evidence that prenatal SSRI treatment can induce ASD in humans. Thus, there is ample evidence that excess 5-HT, especially during development, can induce ASD-like symptoms in humans as well as in rodents.

A second explanation could be that perhaps the interaction between VPA and enhanced 5-HT levels is relevant for the core but not the auxiliary symptoms of ASD. Although this is possible, and we are currently evaluating the interaction model in relation to social behavior, communication and stereotypy, we specifically focussed on cognition and anxiety in this paper because of the clear link with 5-HT. Indeed, homozygous SERT KO rats and mice show increased anxiety-like behavior (Holmes et al., 2003; Olivier et al., 2008), as do rats and mice prenatally treated SSRIs (Olivier et al., 2011). Although less well studied, there is also evidence for cognitive alterations in (homozygous) SERT knock-out rats (Nonkes and Homberg, 2013), including a reduction in LI (Nonkes et al., 2012). Considering the notorious heterogeneity of ASD symptomatology in humans and the fact that cognitive and anxiety symptoms (in contrast to the core deficits) are not seen in all patients with ASD (Levy et al., 2009), it is still possible that we may find significant interactions with other aspects of ASD.

A third possible explanation for failing to find a significant gene–environment interaction would be the presence of a ceiling or floor effect. Theoretically, if either of the two factors, by themselves, already show a strong effect, a further increase in this effect by the second factor might not be possible. Again, this seems an unlikely explanation given the fact that we purposely used a relatively moderate dose of VPA combined with heterozygous SERT knock-out rats. Moreover, inspection of the figures shows that, when there was a significant effect of VPA treatment, this effect was only moderate, leaving room for a moderation by the genotype.

The final, and perhaps most likely, explanation for the lack of an interaction effect is related to the issue of timing. In the current set of experiments, VPA was injected at GD12, in line with most other studies that have been performed in rodent models relating to ASD (Schneider and Przewlocki, 2005; Roullet et al., 2013; Ranger and Ellenbroek, 2015), and consistent with findings that similar injections on GD7, 9 or 15 were significantly less effective in changing sociability and in seizure threshold (Kim et al., 2011). However, the studies that have investigated alterations in 5-HT neurodevelopment and functioning have all applied VPA at GD9 (Miyazaki et al., 2005; Tsujino et al., 2007; Dufour-Rainfray et al., 2010; Oyabu et al., 2013). Together this might suggest that while in normal animals GD12 is the optimal time for inducing ASD-like symptoms, GD9 might be more optimal for rats with a compromised 5-HT system. Although so far we have no evidence for this hypothesis, it would be in line with multiple other studies showing that the timing of early adverse environmental factors crucially determines the long term outcome. For example, within the human literature, research investigating the effects of the Dutch famine in 1944/1945 (Kyle and Pichard, 2006), of bereavement (Class et al., 2014) and of hurricane exposure (Kinney et al., 2008a) on psychopathology show clear time-dependent effects. Likewise, studies from our own laboratory have shown that the long term consequences of maternal deprivation (Ellenbroek and Riva, 2003) or prenatal exposure to lipopolysaccharide (Waterhouse et al., 2016) critically depend on the timing of the environmental insult. Thus, this leads to the exiting idea that the nature of the gene ^*^ environmental interaction is also governed by the specific timing of the environmental factor. Future research will obviously be necessary to further investigate this hypothesis.

In summary, in the present paper we set out to investigate whether prenatal exposure to VPA at GD12 increased anxiety and disrupted cognitive processes and whether this effect was enhanced in rats with a genetically compromised serotonergic system. We found clear evidence for the first: an increase in anxiety in both the elevated plus maze and the novelty suppressed feeding and a decrease in cognition as evidenced by a reduction in sucrose intake in the latent inhibition task and in prepulse inhibition. However, we failed to find strong support for a gene–environment interaction and we have discussed that this may be related to the timing of the VPA injection.

## Author contributions

BE was involved in the planning of the experiments, analysing the data and wrote the majority of the paper. CA performed the experiments, analyzed the data and was involved in the writing of the paper. JY was involved in the analysis of the data and in writing the paper.

### Conflict of interest statement

The authors declare that the research was conducted in the absence of any commercial or financial relationships that could be construed as a potential conflict of interest.
